# Sympatric occurrence of *Ixodes ricinus* with *Dermacentor reticulatus* and *Haemaphysalis concinna* and the associated tick-borne pathogens near the German Baltic coast

**DOI:** 10.1186/s13071-022-05173-2

**Published:** 2022-02-22

**Authors:** Cristian Răileanu, Oliver Tauchmann, Cornelia Silaghi

**Affiliations:** 1 Institute of Infectology, Friedrich-Loeffler-Institut, Suedufer 10, 17493 Greifswald-Insel Riems, Germany; 2 Department of Biology, University of Greifswald, Domstraße 11, 17489 Greifswald, Germany

**Keywords:** Ixodid ticks, Tick-borne pathogens, Co-infections, German Baltic coast

## Abstract

**Background:**

Ixodid ticks from the Northern Hemisphere have registered a northward expansion in recent years, and *Dermacentor reticulatus* is such an example in Europe, its expansion being considered a result of climate change alongside other factors. The aim of this study was to identify the composition of questing tick species and the associated pathogens at different sites near the German Baltic coast.

**Methods:**

Questing ticks were collected monthly at four sites (May–November, 2020), mainly grasslands, and in October and November 2020 at a fifth site. Molecular screening of ticks for pathogens included RT-qPCR for the tick-borne encephalitis virus (TBEV), qPCR for *Anaplasma phagocytophilum,* PCR for *Babesia* species and *Rickettsia* species, and nested PCR for *Borrelia* species.

**Results:**

Altogether 1174 questing ticks were collected: 760 *Ixodes ricinus*, 326 *D. reticulatus* and 88 *Haemaphysalis concinna*. The highest activity peak of *I. ricinus* and *D. reticulatus* was in May, in June for *H. concinna* while a second peak was observed only for *I. ricinus* and *D. reticulatus* in September and October, respectively. All samples tested negative for TBEV. For *A. phagocytophilum*, 1.5% of *I. ricinus* adults tested positive while the minimum infection rate (MIR) in nymphs was 1.3%. This pathogen was found in 0.6% of *D. reticulatus*. *Babesia* spp. were detected in *I. ricinus* (18.2% adults, 2.1% MIR in nymphs) and *H. concinna* (13.3% adults, 9.7% MIR in nymphs). *Borrelia* spp. were present only in *I. ricinus* (49.1% adults, 11.9% MIR in nymphs), while *Rickettsia* spp. were detected in *I. ricinus* (14% adults, 8.9% MIR in nymphs) and *D. reticulatus* (82%). Co-detection of pathogens was observed in 26.6% and 54.8% of positive *I. ricinus* adults and nymph pools, respectively, while one *D. reticulatus* tested positive for *A. phagocytophilum* and *Rickettsia* spp. The most common co-infection in *I. ricinus* adults was *Babesia microti* and *Borrelia afzelii* (12.3% of positive ticks).

**Conclusions:**

The results of this study confirm the northern expansion of *D. reticulatus* and *H. concinna* in Germany. The detailed data of the infection levels at each location could be useful in assessing the risk of pathogen acquisition following a tick bite.

**Graphical Abstract:**

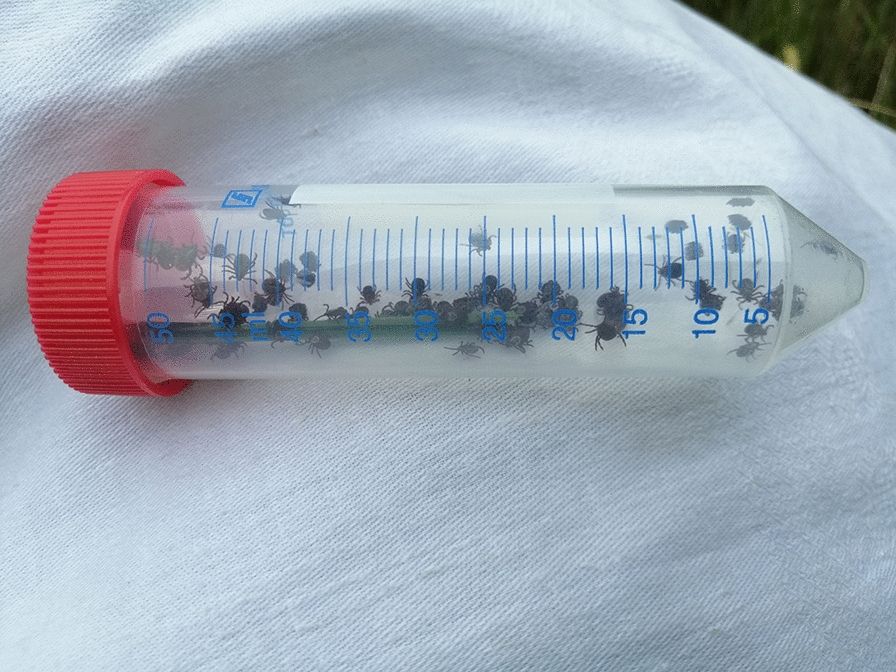

**Supplementary Information:**

The online version contains supplementary material available at 10.1186/s13071-022-05173-2.

## Background

The ixodid tick fauna in Germany is represented by a total of 19 species [1]. In addition to these, two tick species of the exotic *Hyalomma* genus (*Hyalomma marginatum* and *Hyalomma rufipes*) have been recently reported in Germany alongside their ability to develop from nymphs to adults under the local climatic conditions [2]. Another imported species in Germany, *Rhipicephalus sanguineus* sensu lato (s.l.) was found on dogs after travellers returned from regions with a tropical or subtropical climate. Additionally, it is considered that this tick species can survive indoors during the winter [1].

The geographic range of *Ixodes ricinus* extends on longitude from 10° W (Ireland) to 45° E (Ural Mountains, Russia) and on latitude from 60° N (Sweden) to 30° N (Egypt) [3]. It is the most common tick species in Germany, and is present in all 16 federal states [1]. One of the main characteristics supporting the wide geographic distribution of this tick species is the low host specificity. More than 300 vertebrate species are described as feeding hosts for *I. ricinus* ([4] after Bowmann and Nuttall, 2008). It is an exophilic tick species with three different developmental stages, each stage feeding on different hosts to continue the life cycle. *Ixodes ricinus* is mainly found in deciduous and mixed forests and shrubs but also in urban and peri-urban habitat types [4]. It can transmit a broad spectrum of pathogens to both animals and humans, with the tick-borne encephalitis virus (TBEV) and *Borrelia burgdorferi* sensu lato (s.l.). being among the most relevant disease agents in Europe for public health. *Ixodes ricinus* is considered the best studied tick species in Germany, and various reports have confirmed its ability to carry multiple pathogens (bacteria, viruses or parasites) [1, 5–10].

*Dermacentor reticulatus* is the second most commonly encountered tick species in Central Europe after *I. ricinus,* having a distribution that extends from 9° W (Portugal) to 88° E (western Siberia) and within the latitude range of 34–60° N [11]. The geographic range of this species in Germany indicates that it is widespread in the eastern federal states of Brandenburg, Saxonia, Saxonia-Anhalt and Berlin [1]. The northern location of *D. reticulatus* in Germany is represented by a site at the Baltic Sea coast in the port of Rostock [11] and there are also indications needing confirmation that its range extends further north, a citizen science project reporting the presence of *D. reticulatus* on the island of Sylt [12]. Similar to *I. ricinus*, it is a three-host tick species, and while adults are exophilic ticks, feeding on large mammals (dogs, sheep, goats, horses, cattle, occasionally humans), the larvae and nymphs of *D. reticulatus* are nidicolous, feed mostly on rodents and are rarely collected by flagging [13, 14]. The preferred habitats of this tick species are deciduous and mixed forests, meadows and pastures, requiring moderate humidity levels [14]. *Dermacentor reticulatus* is a competent vector for several pathogens such as *Babesia canis* (responsible for canine babesiosis in Europe), *Babesia caballi* (causing equine babesiosis) [15], *Rickettsia raoultii* (causative agent of tick-borne lymphadenopathy) or *Francisella tularensis* (tularaemia) and *Coxiella burnetii* (Q fever agent) [13]. The TBEV has also been detected in *D. reticulatus* from Germany and this tick species is considered to act as a vector for the virus [16].

The west–east distribution of *Haemaphysalis concinna* extends from the Spanish Atlantic coast to Kamchatka (Russia) while the latitude range is 28–64° N [17]. The northern limit of *H. concinna* in Europe is reported from Germany at 53.30° N latitude [18]. In connected water landscapes from Central Europe (such as the Mecklenburg lake plateau in the north-east of Germany or alongside the Danube and Morava rivers), it is considered the third most encountered tick species after *I. ricinus* and *D. reticulatus* [17]. The occurrence of *H. concinna* in Germany is patchy and a total of 24 known locations have been described [1].

The life cycle of *H. concinna* requires three different hosts (small or medium-sized mammals, birds or reptiles). It is an exophilic tick species that completes its developmental cycle within 3 years [19, 20], and it can be found in different habitat types such as deciduous and mixed forests, river valleys, or in close proximity to lakes, in habitats of forest steppes and wet steppes [17, 19]. A broad variety of known tick-borne pathogens have been detected in this tick species such as *B. burgdorferi* s.l. (causative agent of Lyme borreliosis), *C. burnetii, F. tularensis, Babesia divergens* (responsible for bovine babesiosis)*, B. canis,* or *Anaplasma phagocytophilum* (human granulocytic anaplasmosis and tick-borne fever of ruminants) (reviewed by [17]).

The northern distribution limit of *I. ricinus* has been advancing northwards in Sweden since the climate began to change significantly in the late 1980s [21]. In addition, the expansion of *D. reticulatus* in Europe is considered a result of climate change alongside other factors such as the increase of deer abundance and the availability of more fallow land due to the EU agricultural policies [22]. It is essential to conduct active monitoring for an accurate determination of the geographic distribution of ticks in order to rightly assess the risk posed by these ectoparasites in different regions. In this study, a collection of questing ticks was conducted to identify the composition of tick species and the associated pathogens at different sites near the German Baltic coast, north-east of Germany.

## Methods

### Collection of ticks and species identification

**Fig. 1:**
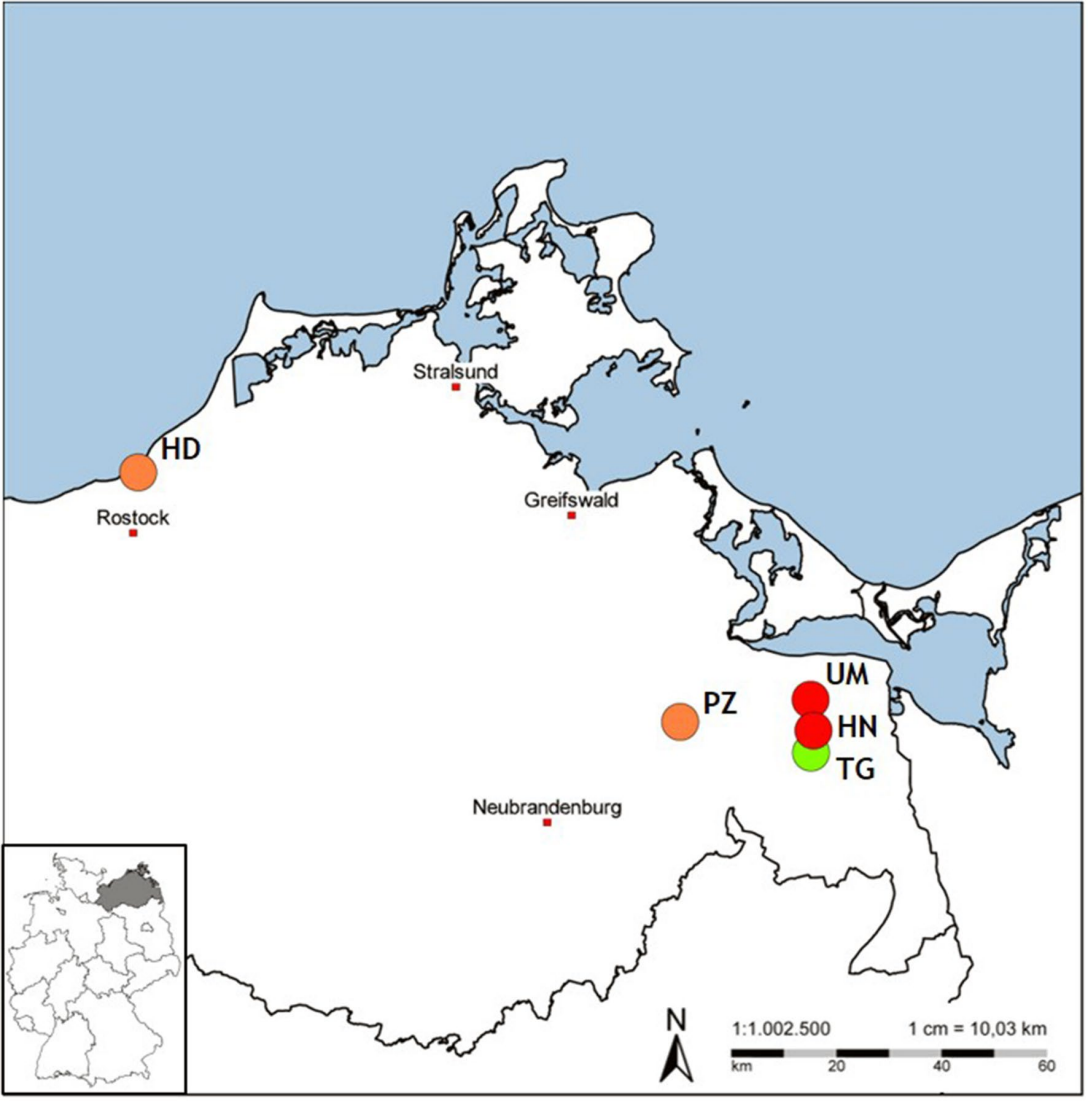
Sites in north-east of Germany from which questing ticks were collected. HD, Hohe Düne; UM, Ueckermünde; HN, Holländerei; TG, Torgelow; PZ, Putzar. Orange circle: sites where *Ixodes ricinus* and *Dermacentor reticulatus* were found; red circle: sites where *I. ricinus* and *Haemaphysalis concinna* were observed; green circle: site where *I. ricinus, D. reticulatus* and *H. concinna* were observed. The map is copyright free and it was built using Map Explorer version 2.0^®^ 2010, FLI, Wusterhausen

Questing ticks were collected from five different locations in Western Pomerania, Germany (Fig. 1). Ticks were collected once a month from May until November 2020 by flagging method [23] between 09:00 am and 02:00 pm at four sites represented mainly by grassland and meadows located in the proximity of lakes or forest: Torgelow (TG; 53.643406, 14.034056), Ueckermünde (UM; 53.733800, 14.043847), Holländerei (HN; 53.680698, 14.046079), and Hohe Düne (HD; 54.188678, 12.135170). These locations were selected after they were evaluated as suitable for encountering *D. reticulatus,* which was the target species to collect in this study. One additional site was included (Putzar-PZ; 53.711169, 13.664667) due to the high tick infestation of a dog reported by the owner, and it was visited in October and November 2020. Ticks were further identified to species and developmental stages using morphological keys [24]. After identification, the samples were stored at −80 °C until further processing.

### Molecular screening

#### DNA and RNA extraction

Adult ticks were processed individually while larvae and nymphs were pooled at a maximum of 10 ticks per pool. One pool consisted of ticks from the same tick species, location and date. Homogenization of ticks was done as previously described [25] while DNA and RNA extractions were performed from 100 μl aliquots by using NucleoMag^®^ VET kit (Macherey–Nagel, Düren, Germany) and the KingFisher^®^ Flex purification system (Thermo Fisher, Darmstadt, Germany), following the manufacturer’s instructions. Total DNA and RNA were eluted in 100 μl of elution buffer and stored at −80 °C until further use.

#### Polymerase chain reaction (PCR) and nested PCR for tick species identification and pathogen detection

A total of 59 ticks morphologically identified as *I. ricinus* (*n* = 40, representing 7.4% of females, 7.6% of males and 11.4% of the pool of nymphs), *H. concinna* (*n* = 8) and *D. reticulatus* (*n* = 11) were included in a PCR for genetic confirmation of the tick species based on a part of the cytochrome c oxidase subunit I (*COX1*) gene. PCR was also conducted for all tick samples to detect *Babesia*/*Theileria* spp. *18S rRNA* gene and *Rickettsia* spp. *gltA* gene, while a nested PCR was done to detect *Borrelia* spp. 16S–23S rRNA intergenic spacer (IGS). PCR for the *Rickettsia* *ompA* gene and nested PCR for *Rickettsia* *ompB* gene were done only for a part of the samples that tested positive after *Rickettsia* gltA PCR in the attempt to identify the *Rickettsia* species: 43 *D. reticulatus* samples tested for *Rickettsia* ompA*,* 57 *I. ricinus* and 43 *D. reticulatus* samples tested for *Rickettsia* ompB. The reactions were performed using the GoTaq^®^ Flexi DNA Polymerase Kit (Promega, Walldorf, Germany) and a C1000 thermal cycler (Bio-Rad Laboratories Inc., Feldkirchen, Germany). The amplification conditions and primer concentrations were followed as stated in the available publications for each target (Table 1). Each reaction included a positive control (DNA from ticks) and a negative control (molecular-grade water). The PCR products were separated on a 1.5% agarose gel stained with ROTI^®^-GelStain Red (Carl Roth GmbH, Karlsruhe, Germany), then visualized using the ChemiDoc™ MP Imaging System (Bio-Rad Laboratories, Hercules, CA, USA).

**Table 1:** Primers used for tick species confirmation and detection of pathogens in ticks

Target gene	Reaction	Sequence (5′–3′)	Amplicon size (base pairs)	Annealing	Reference
Tick species identification					
* COX1*	PCR	cox1F: GGAACAATATATTTAATTTTTGG cox1R: ATCTATCCCTACTGTAAATATATG^a^	849	55 °C	[51]
*Babesia/Theileria* spp.					
* 18S rRNA*	PCR	BJ1: GTCTTGTAATTGGAATGATGG BN2: TAGTTTATGGTTAGGACTACG^a^	411–452	55 °C	[52]
*Rickettsia* spp.					
* gltA*	PCR	Rsfg877: GGGGGCCTGCTCACGGCGG Rfsg1258: ATTGCAAAAAGTACAGTGAACA^a^	381	56 °C	[53]
* ompA*	PCR	Rr190.70p: ATGGCGAATATTTCTCCAAAA Rr190.701n: GTTCCGTTAATGGCAGCATCT^a^	631	46 °C	[54]
* ompB*	Nested PCR	PCR1: ompB-OF: GTAACCGGAAGTAATCGTTTCGTAA ompB-OR: CTTTATAACCAGCTAAACCACC Nested PCR: ompB SFG-IF: GTTTAATACGTGCTGCTAACCAA^a^ ompB SFG/TG-IR: GGTTTGGCCCATATACCATAAG^a^	489 425	54 °C 56 °C	[55]
*Borrelia* spp.					
* 16S-23S rRNA*	Nested PCR	PCR1: Bospp-IGS-F: GTATGTTTAGTGAGGGGGGTG Bospp-IGS-R: GGATCATAGCTCAGGTGGTTAG Nested PCR: Bospp-IGS-Fi: AGGGGGGTGAAGTCGTAACAAG Bospp-IGS-Ri: GTCTGATAAACCTGAGGTCGGA^a^	1007 388–685	56 °C 58 °C	[56]
*Anaplasma phagocytophilum*					
Major surface protein 2 (* Msp2*)	qPCR	ApMSP2f: TGGAAGGTAGTGTTGGTTATGGTATT ApMSP2r: TTGGTCTTGAAGCGCTCGTA ApMSP2p: TGGTGCCAGGGTTGAGCTTGAGATTG	77	60 °C	[57]
Tick-borne encephalitis virus					
3′non-coding region	RT-qPCR	F_TBE_1: GGGCGGTTCTTGTTCTCC R_TBE_1: ACACATCACCTCCTTGTCAGACT TBE-probe-WT: TGAGCCACCATCACCCAGACACA	67	60 °C	[58]

^a^Primers used in sequencing PCR

#### qPCR for *Anaplasma phagocytophilum*

Tick samples were screened for the *A. phagocytophilum msp2* gene using specific primers and probe (Table 1). The fluorogenic probe included a 6-carboxyfluorescein (FAM) reporter attached to the 5′ end and a Black Hole Quencher 1 (BHQ1) at the 3′ end. The reaction was performed using iTaq™ universal probes supermix (Bio-Rad Laboratories, Inc., Munich, Germany) and CFX-96 Real-Time system (Bio-Rad Laboratories, Inc., Munich, Germany). The reaction mix included 10 µL of DNA, 12.5 µL of iTaq supermix (2×), 900 nM of each primer, 120 nM probe and 1.75 µL of nuclease-free water in a total volume of 25 µL. The initial denaturation step at 95 °C for 5 min was followed by 40 cycles of denaturation at 95 °C for 5 s and annealing/elongation at 60 °C for 30 s. Each reaction included positive (DNA of positive ticks) and negative (molecular grade water) controls.

#### RT-qPCR for detection of tick-borne encephalitis virus

Tick RNA samples were screened for TBEV by RT-qPCR targeting the 3′ non-coding region of the TBEV genome with specific primers and probe (Table 1). The reactions were done in a final volume of 20 μl using the iTaq™ Universal Probes One-Step Kit (Bio-Rad Laboratories, Inc., Munich, Germany). Each assay contained 5 μl of RNA, 10 μl iTaq universal probes reaction mix (2×), 400 nM for each forward and reverse primers and 200 nM probe, 0.5 μl iScript advanced reverse transcriptase and 2 μl of water. Each run included RNA extracted from TBEV Neudoerfl viral stock as positive control and water as negative control. Thermal cycling conditions were as follows: 50 °C for 10 min, 95 °C for 5 min, 45 cycles at 95 °C for 15 s then 60 °C for 1 min.

### Sequencing the PCR products

The sequencing strategy included the samples that tested positive for more than one pathogen after the molecular analysis. In addition to those, 13 *D. reticulatus* samples were also sequenced based on the *gltA* gene to identify the *Rickettsia* species after testing positive only for this pathogen. In the attempt to clearly determine the *Rickettsia* species, the 13 samples that were sequenced for *Rickettsia* spp. *gltA* and 30 additional *D. reticulatus* samples were further sequenced based on the *ompA* and *ompB* genes. Two *H. concinna* samples positive only for *Babesia*/*Theileria* spp. were also sequenced.

The PCR products were purified with NucleoSEQ^®^ kit (Macherey–Nagel, Düren, Germany), following manufacturer’s instructions. After purification, samples were included in a PCR assay, in a 10 µL reaction mix: 1 µL of 5× Sequence buffer, 2 µL BigDye Ready Reaction Mix (Thermo Fischer, Darmstadt, Germany), 1 µL of the reverse primer (10 µM), 5 µL of molecular-grade water and 1 µL of the purified PCR product. The thermal conditions included a denaturation step at 96 °C for 1 min, then 25 cycles of denaturation at 96 °C for 10 s, annealing at the specific temperature for each primer for 5 s (Table 1), elongation at 60 °C (duration depending on the size of the product). The obtained products were again purified with NucleoSEQ kit (Macherey-Nagel, Düren, Germany) and 15 µL of each purified product were mixed with 15 µL of highly deionized (Hi-Di) formamide in a 1.5 ml tube and sequenced at the Institute of Diagnostic Virology, Friedrich-Loeffler-Institut, Germany.

The obtained sequences were viewed and edited in Geneious Prime 2021.0.1 (https://www.geneious.com) and compared with available sequences from GenBank database using BLASTn (http://www.ncbi.nlm.nih.gov.library.vu.edu.au/BLAST/.).

### Phylogenetic analysis

All sequences obtained for the genetic identification of *D. reticulatus* and *H. concinna* based on a part of the *COX1* gene were used for the construction of phylogenetic trees together with sequences retrieved from GenBank. The alignment of the sequences was conducted in MEGA X using ClustalW [26, 27]. Model test was then run in MEGA X for the selection of the suitable model prior to building the phylogenetic trees.

### Statistical analysis

Statistical differences and 95% confidence intervals (CI) were calculated with GraphPad Prism version 9.0.0 for Windows (GraphPad Software, La Jolla, CA, USA, www.graphpad.com). Ordinary one-way analysis of variance (ANOVA) and Tukey’s multiple comparison tests were performed on adult tick infection rates for different geographic groups, tick species and pathogens. The unpaired *t*-test with Welch’s correction was used to compare the infection levels between two different groups. The results for pathogens detected in pooled samples are expressed as the minimum infection rates (MIR), representing the ratio of the number of positive pools to the total number of analysed ticks, assuming that only one tick from a pool was positive. Differences were considered statistically significant when *P* < 0.05.

## Results

### Identified tick species and their seasonality

A total of 1174 questing ticks were collected from May to November 2020 at five different locations from Western Pomerania, Germany. The morphological identification revealed three different tick species. *Ixodes ricinus* was the most frequently encountered species, with 760 ticks collected (524 adults and 236 nymphs) from all five locations. *Dermacentor reticulatus* was found in three locations (TG, HD and PZ), with 325 adult ticks and one nymph collected. In addition to the above-mentioned tick species, 88 *H. concinna* specimens (15 adults, 62 nymphs and 11 larvae) were found at three collection sites (TG, HN and UM) (Table 2).

**Table 2:** Questing ticks collected at five locations in Western Pomerania in 2020

Tick species	Stage	Collection Sites					Total
		HD	TG	HN	UM	PZ	
*Ixodes ricinus*	A	306	159	3	55	1	524
	N	24	204	1	6	1	236
*Dermacentor reticulatus*	A	116	3	0	0	206	325
	N	0	0	0	0	1	1
*Haemaphysalis concinna*	A	0	8	6	1	0	15
	N	0	61	0	1	0	62
	L	0	11	0	0	0	11

*A* adults, *N* nymphs, *L* larvae, *HD* Hohe Düne, *TG* Torgelow, *HN* Holländerei, *UM* Ueckermünde, *PZ* Putzar

**Fig. 2:**
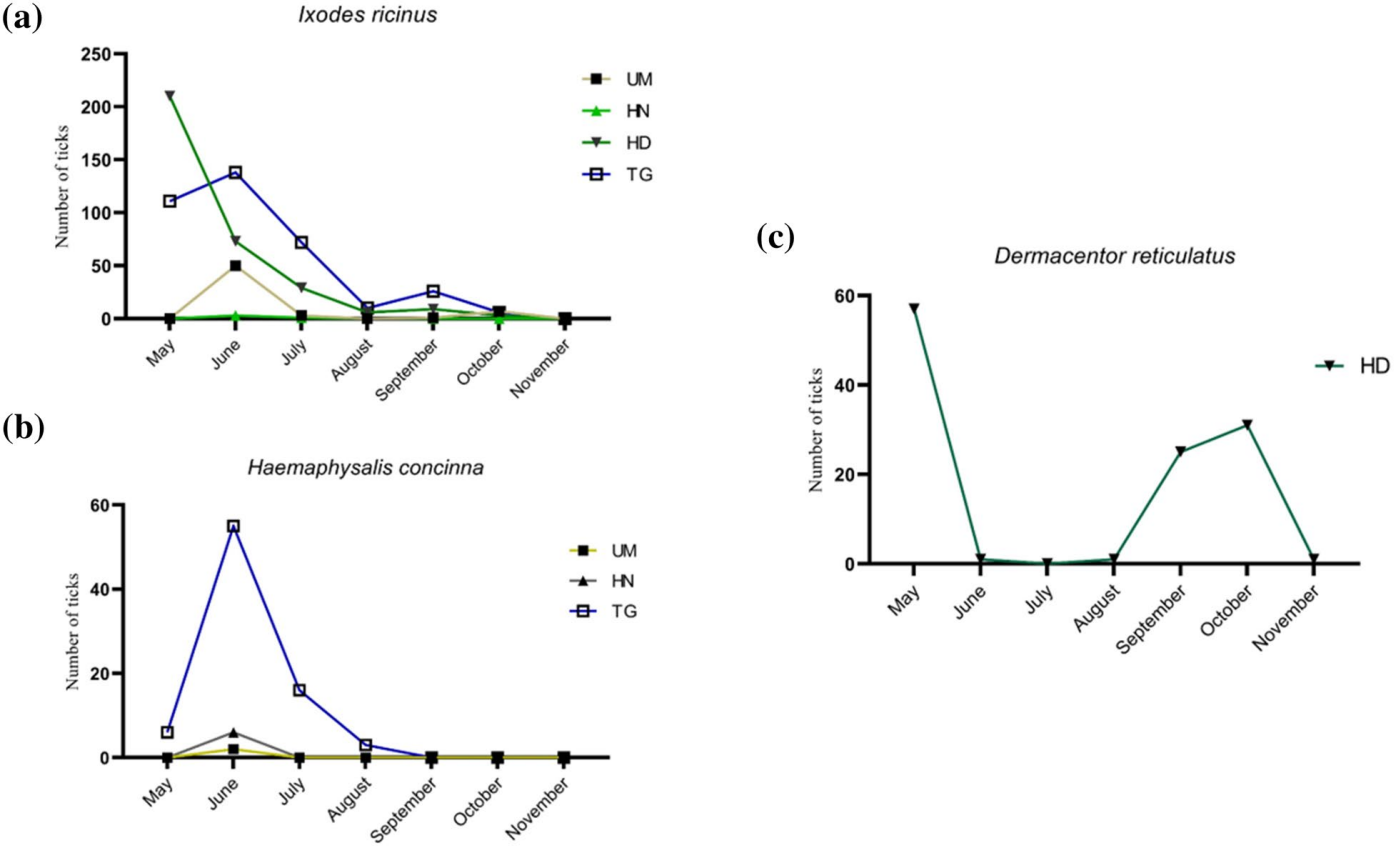
Monthly distribution of collected ticks. **a** *Ixodes ricinus* monthly collected at four different locations: UM, Ueckermünde; HN, Holländerei; HD, Hohe Düne; TG, Torgelow. **b** Monthly records for *Haemaphysalis concinna* at three different locations (UM, HN and TG). **c** *Dermacentor reticulatus* monthly distribution from HD

The monthly distribution of collected ticks was recorded for *I. ricinus* from four locations (TG, UM, HN and HD), for *D. reticulatus* only from the HD site, and for *H. concinna* from three locations (TG, UM and HN) (Fig. 2).

As shown in Fig. 2, *Ixodes ricinus* were mostly found in May and June (77.2% of the total monthly collected ticks) while for *D. reticulatus* the highest number of flagged ticks was in May (49.1% of the monthly collected *D. reticulatus*) followed by a second peak in October (26.7%). *Haemaphysalis concinna* were mostly found in June (71.6%) without registering a second peak of occurrence.

BLAST analysis for the valid sequences of *I. ricinus* (38/40) confirmed the morphological identification of this tick species and had 99.6–100% identity with reference *I. ricinus* sequences from GenBank (AY945440 and KF197122). Molecular identification of selected *D. reticulatus* ticks was successful for all samples (*n* = 11) and BLAST analysis showed that the sequences from this study are 100% identical to *D. reticulatus* reference sequences (AF132829). The molecular confirmation was done also for *H. concinna* samples and the sequences (*n* = 8) matched 96.6–99.2% with *H. concinna* sequences from GenBank (KR108858).

**Fig. 3:**
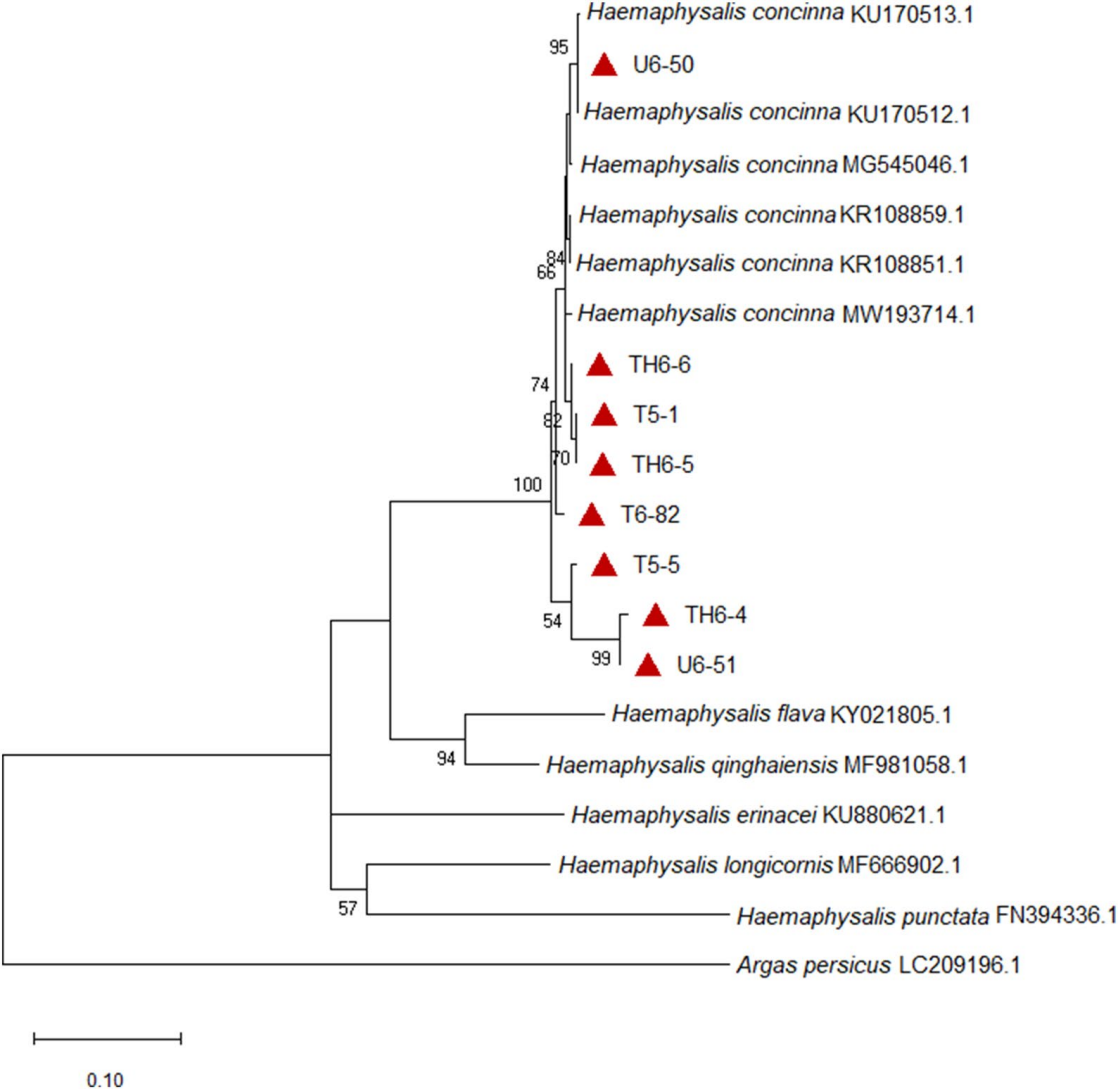
Phylogenetic tree based on a part of the *COX1* gene of *Haemaphysalis concinna*. The tree was constructed using the maximum likelihood method and Tamura–Nei model [50] with bootstrap support of 1000 replicates. The bootstrap values lower than 50 were omitted from the tree. *Argas persicus* sequence (GenBank access. no.: LC209196.1) served as outgroup. Entries preceded by a red triangle represent the sequences from the current study: samples from site UM: U6-50 and U6-51; samples from site HN: TH6-4, TH6-5 and TH6-6; samples from site TG: T5-1, T5-5 and T6-82. Low bootstrap values (usually < 50) should be omitted from the tree.

**Fig. 4:**
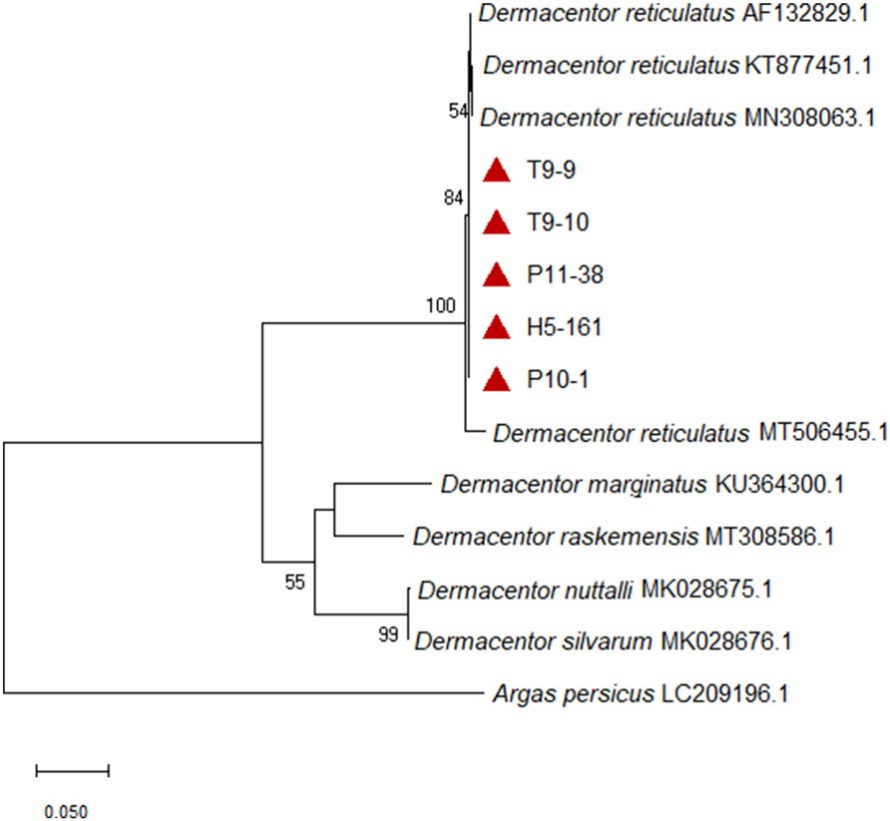
Phylogenetic tree based on a part of the *COX1* gene of *Dermacentor reticulatus*. The tree was constructed using the maximum likelihood method and Tamura–Nei model [50] with bootstrap support of 1000 replicates. The bootstrap values lower than 50 were omitted from the tree. *Argas persicus* sequence (GenBank access. no.: LC209196.1) was used as outgroup. Entries marked with a red triangle represent the sequences from the current study: ticks from site TG: T9-9 and T9-10; ticks from site PZ: P10-1 and P11-38 and sample H5-161 from site HD. Low bootstrap values (usually <50) should be omitted from the tree.

The phylogenetic analysis based on the *COX1* gene confirmed the morphological and molecular identification of *D. reticulatus* and *H. concinna*, the isolates from this study positioned within the same clades with the reference sequences retrieved from GenBank for the corresponding species (Figs. 3, 4).

### Molecular screening of tick-borne pathogens

Out of the total number of collected ticks, 964 were screened for the selected pathogens: 714 *I. ricinus* (479 adults and 235 nymphs), 162 *D. reticulatus* (161 adults and one nymph) and 88 *H. concinna* (15 adults, 62 nymphs and 11 larvae)*.* The RNA samples of all ticks tested negative for the specific RNA of TBEV. Table 3 includes detailed data regarding the prevalence rates for each pathogen identified in different tick species and developmental stages.

**Table 3:** Number of positive ticks and prevalence rates by pathogen, tick species and collection site

Location	Tick species	Stage	Total tested (*n*)	*Babesia*/*Theileria* spp.			*Rickettsia* spp.			*Borrelia* spp.			*A. phagocytophilum*		
				Positive (*n*)	%	95% CI	Positive (*n*)	%	95% CI	Positive (*n*)	%	95% CI	Positive (*n*)	%	95% CI
HD	*I. ricinus*	A	262	69	26.3	21.4–32.0	28	10.7	7.5–15.0	152	58.0	52.0–63.8	1	0.4	0.02–2.1
		N^a^	24	2	8.3	1.5–25.8	2	8.3	1.5–25.8	6	25.0	12.0–44.9	0	0	0
	*D. reticulatus*	A	73	0	0	0	63	86.3	76.6–92.4	0	0	0	1	1.4	0.1–7.4
TG	*I. ricinus*	A	159	10	6.3	3.5–11.2	34	21.4	15.7–28.4	55	34.6	27.6–42.3	5	3.1	1.4–7.1
		N^a^	204	3	1.5	0.4–4.2	16	7.8	4.9–12.4	20	9.8	6.4–14.7	3	1.5	0.4–4.2
	*D. reticulatus*	A	3	0	0	0	3	100	43.9–100	0	0	0	0	0	0
	*H. concinna*	A	8	2	25.0	4.4–59.1	0	0	0	0	0	0	0	0	0
		N^a^	61	6	9.8	4.6–19.8	0	0	0	0	0	0	0	0	0
		L^a^	11	0	0	0	0	0	0	0	0	0	0	0	0
UM	*I. ricinus*	A	55	8	14.5	7.6–26.2	5	9.1	3.9–19.6	28	50.9	38.1–63.6	1	1.8	0.1–9.6
		N^a^	6	0	0	0	2	33.3	5.9–70.0	1	16.7	0.9–56.4	0	0	0
	*H. concinna*	A	1	0	0	0	0	0	0	0	0	0	0	0	0
		N^a^	1	0	0	0	0	0	0	0	0	0	0	0	0
HN	*I. ricinus*	A	3	0	0	0	0	0	0	0	0	0	0	0	0
		N^a^	1	0	0	0	1	100	5.1–100	1	100	5.1–100	0	0	0
	*H. concinna*	A	6	0	0	0	0	0	0	0	0	0	0	0	0
PZ	*D. reticulatus*	A	85	0	0	0	66	77.6	67.7–85.2	0	0	0	0	0	0
		N^a^	1	0	0	0	1	100	5.1–100	0	0	0	0	0	0
Total	*I. ricinus*	A	479	87	18.2	15.0–21.9	67	14.0	11.2–17.4	235	49.1	44.6–53.5	7	1.5	0.7–3.0
		N^a^	235	5	2.1	0.9–4.9	21	8.9	5.9–13.3	28	11.9	8.4–16.7	3	1.3	0.3–3.7
	*D. reticulatus*	A	161	0	0	0	132	82.0	75.3–87.2	0	0	0	1	0.6	0.03–3.4
		N^a^	1	0	0	0	1	100	5.1–100	0	0	0	0	0	0
	*H. concinna*	A	15	2	13.3	2.4–37.9	0	0	0	0	0	0	0	0	0
		N^a^	62	6	9.7	4.5–19.5	0	0	0	0	0	0	0	0	0
		L^a^	11	0	0	0	0	0	0	0	0	0	0	0	0

*HD* Hohe Düne, *TG* Torgelow, *HN* Holländerei, *UM* Ueckermünde, *PZ* Putzar, *A* adults, *N* nymphs, *L* larvae

^a^The results are calculated as minimum infection rate (MIR); %, prevalence registered in ticks

*Ixodes ricinus* ticks tested positive for all other selected pathogens. *Borrelia* spp. was the most encountered pathogen in *I. ricinus* adults (49.1%), showing a significant difference when compared to the infection levels of *Rickettsia* spp. (14.0%), *Babesia/Theileria* spp. (18.2%) or *A. phagocytophilum* (1.5%) (*P* < 0.001). No significant difference was observed between the prevalence rates of *Rickettsia* spp. and *Babesia/Theileria* spp. (*P* = 0.267) but both pathogens had a significantly higher prevalence compared to *A. phagocytophilum* (*P* < 0.001).

From a total of 92 *I. ricinus* samples positive for *Babesia/Theileria* after PCR, 70 samples were positive for more than one pathogen and were sequenced. BLASTn analysis identified 62 sequences similar to *Babesia microti* (MW554613) (57 showed 99.1–100% similarity and five matched 96.3 to 98.7%) while eight samples matched 100% to *Babesia* sp. (MW554616).

Out of 263 *I. ricinus* tick samples positive for *Borrelia,* 114 were sequenced, resulting in a total of 92 valid sequences. The BLASTn search determined 71 sequences as *Borrelia afzelii* (MK945792) (98.4–100% identity)*,* 18 sequences matched 99.3–100% to both *B. afzelii* (MK945782) and *Babesia spielmanii* (JX448323), without a clear distinction between the two genospecies, while the remaining three samples matched 99.9% to *B. burgdorferi* sensu stricto (s.s.) (CP077727), 100% to *Borrelia miyamotoi* (LC540659) and 99.6% to *Borrelia valaisiana* (MK945840)*,* respectively.

Confirmation of *Rickettsia* species was attempted for 57 samples that showed double or triple infection, out of the total 88 positive *I. ricinus* ticks. In total, 51 valid *gltA* sequences were obtained. No clear identification of *Rickettsia* species was possible, 44 samples had 99.4–100% identity to *R. helvetica* (GenBank accession number: MH618386) and uncultured *Rickettsia* sp. (KX051405), three sequences showed 99.4 and 100% identity to *R. raoultii* (MT293352) or *Rickettsia* sp. (MT424977), two samples had 99.4 and 100% identity to *R. monacensis* (MH618388) and uncultured *Rickettsia* (LC060719), and two sequences matched 100% to *Candidatus* Rickettsia mendelii (KJ882309) and uncultured *Rickettsia* sp. (AB911109).

*Rickettsia* ompB sequencing was successful for 49 samples, out of which 44 matched 100% to *R. helvetica* (KY951985) and one had 99.7% similarity to *R. helvetica* (LC461080). For the remaining four samples, clear identification was not possible. Detailed data regarding the results retrieved after BLAST analysis of *Rickettsia* sequences are available in Additional file 1.

The second most commonly encountered tick species, *D. reticulatus,* tested positive for *Rickettsia* spp. and *A. phagocytophilum*. The infection rate of *Rickettsia* spp. in adults (82.0%; 133/162) was significantly higher compared to *A. phagocytophilum* (0.6%) (*P* < 0.001). Due to the high prevalence of *Rickettsia* spp. in *D. reticulatus*, 13 samples positive only for *Rickettsia* spp. were initially sequenced based on *gltA* gene. Sequencing results for *gltA* gene did not result in a clear identification of *Rickettsia* species, 2 samples had 100% identity to *Rickettsia helvetica* (GenBank accession number: MH618386) and uncultured *Rickettsia* sp. (KX051405), while 11 samples had 99.6–100% identity to *R. raoultii* (MN388798) or uncultured *Rickettsia* sp. (MN431836). Species confirmation was attempted for the 13 samples and 30 additional ones based on *ompA* and *ompB* genes. The results retrieved 40 valid sequences of *Rickettsia* ompA that showed 99.5–100% (*n* = 32 isolates), 98–99.4% (*n* = 7 isolates) and 97.6% (*n* = 1 isolate) similarity to several *R. raoultii* sequences (MF166732, KX506737, JN398480) and one entry identified as *Rickettsia* sp. (AH009131). A distinction between these entries was impossible. In total, 42 *Rickettsia* ompB sequences were obtained and all showed 100% identity to different *R. raoultii* isolates from GenBank (HQ232278, DQ365797, KU310593) and to one entry, *Candidatus* Rickettsia rioja (GQ404431) (Additional file 1).

One *D. reticulatus* tick that showed co-infection with *A. phagocytophilum* and *Rickettsia* spp. was sequenced based on the *gltA* and *ompB* genes to determine the *Rickettsia* species. The *gltA* sequence showed 100% identity to *R. raoultii* (MT293352), *Rickettsia* sp. (MT424977) or uncultured *Rickettsia* sp. (MN431836). Based on a part of the *ompB* gene, the obtained sequence had 100% similarity to *R. raoultii* (HQ232278) and *C.* Rickettsia rioja (GQ404431). The PCR reaction for *Borrelia* spp. and *Babesia/Theileria* spp. showed unspecific amplification and the samples were considered negative.

*Haemaphysalis concinna* ticks tested positive for *Babesia/Theileria* spp., adults showing a prevalence of 13.3%. From the two samples that were sequenced, one matched 100% to *Theileria* sp. (MG214907) and *Theileria capreoli* (MW531681) while the second sample had 99.74% identity to *Babesia* sp. (KU550694).

### Prevalence of pathogens in ticks from different sites

The PCR for *Babesia/Theileria* spp. retrieved positive ticks from all locations except the samples collected from HN (Holländerei) (Table 3). When comparing the *Babesia/Theileria* spp. infection rates of *I. ricinus* adults according to location, there was a significant difference between ticks from HD and TG (26.3% versus 6.3%; *P* < 0.001) but no statistical difference to samples from UM (*P* = 0.089). *Babesia/Theileria* spp. were detected in *H. concinna* ticks collected only from TG (Table 3).

*Rickettsia* was detected in *I. ricinus* from all locations where this tick species was found. After comparing the infection rates of adult ticks, *I. ricinus* from TG (21.4%) showed a significant difference compared to the samples from HD (10.7%) (*P* = 0.006). The molecular screening detected high infection rates of *D. reticulatus* for *Rickettsia* spp. There was no statistical difference between the infection rates in ticks from sites HD and PZ (*P* = 0.261) while all the three *D. reticulatus* adults found at the TG site tested positive for this pathogen (Table 3).

*Borrelia* was the most encountered pathogen in *I. ricinus* ticks, the statistical analysis in adults indicated a significantly higher prevalence for samples from HD compared to TG (58% vs 34.6%; *P* < 0.001) but no significance when compared to UM site (50.9%) (*P* = 0.591) (Table 3).

*Anaplasma phagocytophilum* was identified in *I. ricinus* ticks from sites HD, TG and UM, with no statistically significant differences among adults from different collection sites (*P* = 0.072) (Table 3). Concerning *D. reticulatus,* only the ticks collected in the HD site were positive for this pathogen, with a 1.4% prevalence of infection. Overall, all the *H. concinna* ticks tested negative for the presence of *A. phagocyophilum* DNA.

### Infection rates of pathogens in different tick species

There was no significant difference of *Babesia/Theileria* spp. overall prevalence in *I. ricinus* adults (18.2%) compared to *H. concinna* ticks (13.3%) (*P* = 0.609) while the prevalence in *H. concinna* at site TG (25%) was higher compared to *I. ricinus* (6.3%) without a statistical significance (*P* = 0.292) (Table 3).

### Co-detection of pathogens

From the total number of *I. ricinus* ticks that tested positive for at least one putative pathogen, 26.6% of adults (78/293) and 54.8% of nymph pools (17/31) showed an association with two or three different pathogens.

In total, 17 different associations were observed in *I. ricinus*: 11 different co-detections of two pathogens and six different associations with three pathogens (Table 4). The most common co-infection in adults was with *B. microti* and *B. afzelii* (12.3% of positive ticks), showing a statistically higher infection rate compared to all other co-infections (*P* < 0.001). No significant difference was observed when comparing the co-infection levels with *B. microti* and *B. afzelii* in *I. ricinus* adults from sites HD, TG or UM. The second most common co-infection in adults was with *B. afzelii* and *R. helvetica* (4.4% of positive ticks).

**Table 4:** Co-detection rates observed in positive ticks for at least one pathogen by location, tick species and developmental stage

	HD			TG					UM		HN		PZ		Total					
	IR		DR	IR		DR	HC		IR	HC	IR	HC	DR		IR		DR		HC	
	A	N	A	A	N	A	A	N	A	N	N	A	A	N	A	N	A	N	A	N
Total positive ticks	**185**	**6**	**63**	**75**	**21**	**3**	**6**	**8**	**33**	**3**	**1**	**5**	**66**	**1**	**293**	**31**	**132**	**1**	**11**	**8**
*B. microti* + *B. afzelii*	13.0	0	0	8.0	4.8	0	0	0	18.2	0	0	0	0	0	12.3	3.2	0	0	0	0
*B. microti* + *Borrelia* sp.	5.4	33.3	0	0	0	0	0	0	0	0	0	0	0	0	3.4	6.5	0	0	0	0
*Babesia* sp. + *B. afzelii*	0.5	0	0	0	4.8	0	0	0	6.1	0	0	0	0	0	1.0	3.2	0	0	0	0
*Babesia* sp*.* + *Borrelia* sp*.*	1.1	0	0	0	0	0	0	0	0	0	0	0	0	0	0.7	0	0	0	0	0
*B. microti* + *R. helvetica*	1.1	0	0	0	0	0	0	0	0	0	0	0	0	0	0.7	0	0	0	0	0
*Babesia* sp*.* + *R. helvetica*	0.5	0	0	0	4.8	0	0	0	0	0	0	0	0	0	0.7	0	0	0	0	0
*Babesia* sp*.* + *Rickettsia* sp*.*	0.5	0	0	0	0	0	0	0	0	0	0	0	0	0	0.3	0	0	0	0	0
*B. afzelii* + *R. helvetica*	3.8	0	0	8	33.3	0	0	0	0	0	100	0	0	0	4.4	25.8	0	0	0	0
*B. valaisiana* + *R. helvetica*	0.5	0	0	0	0	0	0	0	0	0	0	0	0	0	0.3	0	0	0	0	0
*Borrelia spp.* + *R. helvetica*	0.5	16.7	0	0	4.8	0	0	0	0	0	0	0	0	0	0.3	6.5	0	0	0	0
*A. phagocytophilum* + *R. helvetica*	0	0	0	2.7	0	0	0	0	0	0	0	0	0	0	0.7	0	0	0	0	0
*A. phagocytophilum* + *Rickettsia* sp*.*	0	0	1.6	0	0	0	0	0	0	0	0	0	0	0	0	0	0.8	0	0	0
*B. microti* + *B. afzelii* + *R. helvetica*	0.5	0	0	1.3	4.8	0	0	0	0	0	0	0	0	0	0.7	3.2	0	0	0	0
*B. microti* + *B. afzelii* + *R. monacensis*	0.5	0	0	0	0	0	0	0	0	0	0	0	0	0	0.3	0	0	0	0	0
*B. microti* + *B. afzelii* + *Rickettsia* sp*.*	0	0	0	1.3	0	0	0	0	0	0	0	0	0	0	0.3	0	0	0	0	0
*B. microti* + *Borrelia* sp*.* + *R. helvetica*	0.5	0	0	0	0	0	0	0	0	0	0	0	0	0	0.3	0	0	0	0	0
*B. microti* + *Borrelia* sp*.* + *Rickettsia* sp*.*	0.5	0	0	0	0	0	0	0	0	0	0	0	0	0	0.3	0	0	0	0	0
*Babesia* sp*.* + *B. afzelii* + *R. helvetica*	0	0	0	0	4.8	0	0	0	0	0	0	0	0	0	0	3.2	0	0	0	0
Total co-infections	29.2	50	1.6	21.3	61.9	0	0	0	24.2	0	100	0	0	0	26.6	54.8	0.8	0	0	0

*HD* Hohe Düne, *TG* Torgelow, *HN* Holländerei, *UM* Ueckermünde, *PZ* Putzar, *IR* *Ixodes ricinus*, *DR* *Dermacentor reticulatus*, *HC Haemaphysalis concinna*, *A* adults, *N* nymphs

In nymphs, co-detection of *B. afzelii* and *R. helvetica* was observed in 25.8% positive samples followed by the co-detection of *B. microti* and *Borrelia* sp. (6.5%) and *Borrelia* sp. and *R. helvetica* (6.5%).

Co-infection with *A. phagocytophilum* and *Rickettsia* spp. was detected in 0.8% of positive *D. reticulatus* adults (1/132) and it was the only co-infection observed in ticks other than *I. ricinus.* Table 4 contains detailed information on the dual and triple associations of pathogens observed in ticks.

## Discussion

In this study, we conducted a follow-up analysis on the tick population near the Baltic Sea coast to determine the tick species composition and the prevalence rates for some of the most relevant tick-borne pathogens. The results of our previous study, which focused on screening pathogens in *I. ricinus* only, indicated that questing ticks from Mecklenburg-Western Pomerania can harbour several *Borrelia* species and that *B. miyamotoi* infection levels in ticks are at similar levels to other regions in Europe [25]. Considering the current expansion *D. reticulatus* is undergoing alongside other tick species in Germany and other European countries, we aimed to investigate the presence of this species and the potential sympatric occurrence with other ticks at different sites near the coast in north-east Germany.

Overall, we collected and identified three tick species from the assessed areas. As expected, *I. ricinus* was the most frequently encountered tick, previously reported as the species with the widest geographic distribution in Germany [1]. Previous reports indicated the sympatric distribution of *I. ricinus* with *Ixodes inopinatus* in southern and northern Germany [28, 29]. *Ixodes inopinatus* was described in 2014 as a new species closely related to *I. ricinus* [30]. Due to the similar morphological features of *I. ricinus* and *I. inopinatus*, we conducted molecular identification for a representative batch of the collected *I. ricinus* ticks and all sequences belonged to this tick species, confirming the initial morphological identification. Further extensive tick collection campaigns should determine whether *I. inopinatus* is present in Western Pomerania.

*Dermacentor reticulatus* was found at three collection sites which confirms the presence of this species in north-east Germany. In recent years this species continuously expanded its geographic range in several European countries, Germany included, and it is the second most widespread questing tick on the continent after *I. ricinus* [31]. Climate change has been incriminated as the dominant factor for the range expansion of *D. reticulatus*, nevertheless, the importance of other contributing factors like changes in land use, availability of wildlife hosts, and intense travel of humans and their pets should not be neglected [13].

Collecting *D. reticulatus* from the environment in HD site, very close to the German Baltic coast (Fig. 1), confirms the northward expansion of this species in Germany and the previous documentation of this tick in the port of Rostock (12.14°E∕54.15°N) [11]. A recent publication reported the presence of *D. reticulatus* further north, on the island of Sylt. The observation was made during a citizen science, still, further research is needed to confirm this finding [12].

*Haemaphysalis concinna* was the third most prevalent questing tick species collected in this study. The observation of *H. concinna* at site UM (Fig. 1) further expands the reported northern distribution limit of this species in Europe. The previously known limit from Germany was reported in 2016 at the Seedorf military training area (9.26° E/53.30° N) [18]. In this study, *H. concinna* was collected from sites near the Mecklenburg lake plateau, an area that was described as suitable to maintain *H. concinna* tick populations, and where this tick is considered the third most common species after *I. ricinus* and *D. reticulatus* [17]. Information regarding the circulation of *H. concinna* near the German Baltic coast is available since 1960, with published data indicating several specimens collected in 1958 from *Capreolus capreolus* (L.) deer at several locations near Greifswald [32]. Our observations confirm the presence of the *H. concinna* population in the north-east of Germany by providing the exact geographical locations where this species occurs.

*Ixodes ricinus* showed a main peak of occurrence in May–June while *D. reticulatus* was most active in May, followed by a second lower peak in September for *I. ricinus* and September–October for *D. reticulatus.* The occurrence of *H. concinna* suggested only one peak of activity in June. The monthly distribution of occurrence is in line with the known seasonal activity of all three species and could be of use in predicting the seasonality of tick-transmitted diseases [33].

The morphological identification of *D. reticulatus* and *H. concinna* was molecularly confirmed based on the *COX1* gene that proved to be suitable in identifying ticks at species level. The phylogenetic analysis further supported the morphological and genetic species identification, the sequences from this work clustered within the same clade with reference sequences corresponding to *D. reticulatus* and *H. concinna*, respectively, having also good bootstrap support (Figs. 3, 4).

Using molecular methods, ticks were screened to determine the infection rates for some of the most common tick-borne pathogens, using species-specific and generic primers. All samples tested negative for TBEV. Even though there have been recent reports of TBEV RNA detection in ticks [34] and anti-TBEV antibodies in animals [35, 36] from Mecklenburg-Western Pomerania, the results of this study confirm the low risk classification of this region for TBEV. Nevertheless, the northward expansion of ticks, the land use changes that facilitate movement of virus reservoir hosts, and climate change are factors that could facilitate the range expansion of TBEV towards the northern regions of Germany.

*Ixodes ricinus* adults and nymphs were positive for all other analysed pathogens: *Babesia/Theileria* spp., *Rickettsia* spp., *Borrelia* spp. and *A. phagocytophilum*. These results highlight the involvement of this tick species in causing health problems of public and veterinary concern.

The *Babesia/Theileria* spp. prevalence in questing *I. ricinus* adults in our study (18.2%) was higher compared to the observed infection levels in *I. ricinus* from different geographical areas in Germany that ranged between 0.4% and 10.7% [10]. In addition, 70 out of 92 total *Babesia* positive samples were sequenced and most of the isolates (*n* = 62) matched *B. microti*, the protozoan blood parasite responsible for human babesiosis [37]. These results indicate that there is a significant risk of acquiring this protozoan after a tick bite at the sites selected for this work. *Ixodes ricinus* ticks collected at site HD near Rostock showed the highest infection rate for *Babesia* spp. (26.3% in adults and MIR of 8.3% in nymphs), and the transmission of the pathogen can be facilitated also by the high human activity in the vicinity of this collection site.

The observed *Rickettsia* spp. infection rate in *I. ricinus* adults in our study (14%) is higher when compared to data available from questing ticks collected near the German Baltic coast (8.5%) [38], which can suggest an increase over time of the tick infection rates with *Rickettsia* spp. Ticks simultaneously act as vectors and reservoir hosts for *Rickettsia*, and the efficient transovarial transmission in *I. ricinus* ticks observed under laboratory settings might contribute to the maintenance of the microorganism in tick populations [39]. In the sequenced samples, *R. helvetica* was the dominant species (in 45 out of 49 valid sequences). This species belongs to the spotted fever group (SFG) rickettsiae and it is reported as a pathogenic agent in humans transmitted mainly by *Ixodes* ticks [40]. The current study confirms the presence of this pathogen in questing ticks from north-east Germany, yet further analyses are required to provide a clear estimation of *R. helvetica* prevalence in tick populations from this region.

*Borrelia* was the most common pathogen detected in the assessed ticks (49.1% of adult ticks and MIR of 11.9% in nymphs) showing a higher prevalence compared to our previous study (12.1% in adults and MIR of 3.3% in nymphs) [25]. The infection levels of *I. ricinus* ticks for *Borrelia* spp. are also high when compared to other studies in questing ticks from Germany. High infection rates with *B. burgdorferi* s.l. were also reported from southern Germany (up to 36.2%) [41]. The primer pairs used in this study to detect *Borrelia* spp. are also amplifying the 16S–23S rRNA IGS sequence of *B. miyamotoi* relapsing fever agent, and since not all samples were sequenced, the herein results regarding the prevalence of *B. burgdorferi* s.l. should be interpreted with caution. On the other hand, based on the *B. miyamotoi* infection rates found in *I. ricinus* ticks from Europe, ranging from 0.4% in Estonia to 3.5% in France and Germany [42–44], we can expect a high prevalence of *B. burgdorferi* s.l. in *I. ricinus* ticks from this study. *Borrelia* spp. showed high prevalence in *I. ricinus* ticks from sites HD (58% in adults and MIR of 25% in nymphs) and UM (50.9% in adults and MIR of 16.7% in nymphs) (Table 3). These sites are located in peri-urban areas that represent high-risk locations for public health due to increased tick-human contact.

The observed prevalence of *A. phagocytophilum* in *I. ricinus* ticks from this study (1.5% in adults and MIR of 1.3% in nymphs) is comparable to other published data from northern Germany: 1% near the German Baltic Coast [38], 3.6% in Hamburg [45], and 3.8% overall prevalence for a 10-year interval in Hanover [39]. Supplementary research on a larger scale is needed to determine the prevalence of this pathogen in tick populations and furthermore to identify the circulating *A. phagocytophilum* variants which will facilitate the proper evaluation of the risk posed by this tick-borne pathogen to human and animal health.

Molecular screening of *D. reticulatus* ticks for tick-borne pathogens enabled the detection of high infection levels with *Rickettsia* spp. Overall, 82% (132/161) of the adults tested positive with a homogenous distribution of the prevalence among different collection sites. In addition, approximately one third of the positive samples were sequenced for *ompB* gene (42/132) and matched to *R. raoultii* and *C.* Rickettsia rioja. Both of these bacteria are described as causative agents of Dermacentor-borne necrosis erythema and lymphadenopathy (DEBONEL) or tick-borne lymphadenopathy (TIBOLA) in humans [13, 46]. According to these results, the sites at which *D. reticulatus* ticks were observed are locations where people could potentially acquire SFG *Rickettsia* following a tick bite.

From the range of screened tick-borne pathogens, *H. concinna* ticks tested positive only for *Babesia/Theileria* spp., with sequences that matched *Theileria* sp. or *T. capreoli* and *Babesia* sp. Previous studies have reported detection of piroplasmids of the genus *Theileria* in questing *H. concinna* from Austria [47] or Hungary [48], and *Babesia* in *H. concinna* larvae collected from rodents in Slovakia [49], to which the isolate from our study was closely related. These results suggest a potential role of this tick species as vector for *Theileria* and *Babesia* in Central Europe. Vector competence studies would be valuable in understanding the role of *H. concinna* as vector for pathogenic *Theileria* and *Babesia* species.

Co-detection of pathogens was mostly observed in *I. ricinus* ticks; from the adults and nymph pools positive for at least one agent, 26.6% and 54.8% of the respective adults and nymph pools registered association of two or three different pathogens. The results from the pools of nymphs need to be treated with care since they can express an overestimated association of pathogens. Nevertheless, the high number of *I. ricinus* ticks positive for several pathogens confirms the ability of this species to carry a broad range of pathogens, and exhaustive work is needed to fill the gap on the knowledge in regard to the relationship between pathogens (positive or negative interactions) within the same tick.

## Conclusions

In conclusion, the results of our study represent new data that confirm an expansion of the northern distribution limit of *D. reticulatus* and *H. concinna*. We also confirm the existence of suitable habitats for the sympatric occurrence of *I. ricinus, D. reticulatus,* and *H. concinna* near the German Baltic coast, by providing exact geographic locations where these ticks were encountered. The high infection levels of *I. ricinus* with *Babesia/Theileria* spp., *Rickettsia* spp. and *Borrelia* spp., and of *D. reticulatus* with *Rickettsia* spp. at peri-urban areas suggest a high risk for public health that is facilitated also by a potentially increased tick-human contact. We have also confirmed the ability of *I. ricinus* to carry several pathogens and provided detailed data of the infection levels at each location, data that could be useful in assessing the risk of acquiring a pathogen following a tick bite.

## Supplementary Information

**Additional file 1: Table S1.** BLAST results retrieved for *Rickettsia gltA*, *ompA* and *ompB* according to tick species and collection site.

## Data Availability

All data generated or analysed during this study are included in this published article. Newly generated sequences were submitted to the GenBank database and received the following accession numbers: *Babesia* spp.: OL549321-OL549398; *Rickettsia* spp.: OL549399-OL549443; *Borrelia* spp.: OL701405-OL701478; tick species: OL639104-OL639118.

## Abbreviations

MIR: Minimum infection rate
HD: Hohe Düne
UM: Ueckermünde
HN: Holländerei
TG: Torgelow
PZ: Putzar
*COX1*: Cytochrome c oxidase subunit I
*18S** rRNA*: 18 Svedberg ribosomal ribonucleic acid
*gltA*: Citrate synthase gene
*ompA*: Outer membrane protein A
*ompB*: Outer membrane protein B
IGS: Intergenic spacer
*msp2*: Major surface protein 2
BLAST: Basic local alignment search tool
CI: Confidence interval
