# Supplementary material for: Sympatric occurrence of Ixodes ricinus with Dermacentor reticulatus and Haemaphysalis concinna and the associated tick-borne pathogens near the German Baltic coast
Source: Parasit Vectors. 2022 Feb 22;15:65. doi: 10.1186/s13071-022-05173-2 (PMC8862291; doi:10.1186/s13071-022-05173-2)
Supplement: Supplementary file 1 — Additional file 1: Table S1. BLAST results retrieved for Rickettsia gltA, ompA and ompB according to tick species and collection site. [file 13071_2022_5173_MOESM1_ESM.docx]

Table. BLAST results retrieved for *Rickettsia* *gltA*, *ompA* and *ompB* according to tick species and collection site

| **Tick Species** | **Collection site** | ***Rickettsia* *gltA*** | | | ***Rickettsia* *ompA*** | | | ***Rickettsia* *ompB*** | | |
| --- | --- | --- | --- | --- | --- | --- | --- | --- | --- | --- |
|  |  | **Number of sequenced samples** | **Identity**  **(%)** | **BLAST analysis of the valid sequences [n, species, (Access. Nos.)]** | **Number of sequenced samples** | **Identity**  **(%)** | **BLAST analysis of the valid sequences [n, species, (Access. Nos.)]** | **Number of sequenced samples** | **Identity**  **(%)** | **BLAST analysis of the valid sequences [n, species, (Access. Nos.)]** |
| *Ixodes ricinus* | HD | 20 | 100 | 14 *Rickettsia helvetica* (MH618386), Uncultured *Rickettsia* sp. (KX051405) | n.a. | n.a. | n.a. | 17 | 99.1-100 | 13 *R. helvetica* (KY951985) |
|  |  |  |  |  |  |  |  |  |  | 1 *R. helvetica* (MN094827), *Rickettsia* sp. (HM149285) |
|  |  |  | 99.4 | 1 *Rickettsia monacensis* (MH618388), Uncultured *Rickettsia* sp. (LC060719) |  |  |  |  |  | 1 *R. monacensis* (MN094825), Uncultured *Rickettsia* sp. (MF170623)* |
|  | HN | 1 | 100 | 1 *R. helvetica* (MH618386), Uncultured *Rickettsia* sp. (KX051405) | n.a. | n.a. | n.a. | 1 | 100 | 1 *R. helvetica* (KY951985) |
|  | TG | 35 | 99.7-100 | 32 *R. helvetica* (MH618386), Uncultured *Rickettsia* sp. (KX051405) | n.a. | n.a. | n.a. | 34 | 100 | 30 *R. helvetica* (KY951985) |
|  |  |  | 100 | 2 *Candidatus* Rickettsia mendelii (KJ882309), Uncultured *Rickettsia* sp. (AB911109) |  |  |  |  |  | 1 *R. monacensis* (MN094825), Uncultured *Rickettsia* sp. (MF170623)* |
|  |  |  | 99.4 | 1 *R. monacensis* (MH618388), Uncultured *Rickettsia* sp. (LC060719) |  |  |  |  |  | 1 *R. raoultii* (HQ232278, DQ365797, KU310593), *Candidatus* Rickettsia rioja (GQ404431)* |
|  | UM | n.a. | n.a. | n.a. | n.a. | n.a. | n.a. | 1 | 100 | 1 *R. helvetica* (KY951985) |
| *Dermacentor reticulatus* | HD | 6 | 99.7-100 | 4 *Rickettsia raoultii* (MN388798), Uncultured *Rickettsia* sp. (MN431836) | 16 | 99.1-100 | 16 *R. raoultii* (MF166732, KX506737, JN398480), *Rickettsia* sp. (AH009131) | 19 | 100 | 19 *R. raoultii* (HQ232278, DQ365797, KU310593), *C.* Rickettsia rioja (GQ404431)* |
|  |  |  | 100 | 2 *R. helvetica* (MH618386), Uncultured *Rickettsia* sp. (KX051405) |  |  |  |  |  |  |
|  | PZ | 4 | 100 | 4 *R.raoultii* (MN388798), Uncultured *Rickettsia* sp. (MN431836) | 21 | 97.6-100 | 21 *R. raoultii* (MF166732, KX506737, JN398480), *Rickettsia* sp. (AH009131) | 21 | 100 | 20 *R. raoultii* (HQ232278, DQ365797, KU310593), *C.* Rickettsia rioja (GQ404431)* |
|  | TG | 3 | 100 | 3 *R. raoultii* (MN388798), Uncultured *Rickettsia* sp. (MN431836) | 3 | 99.8-100 | 3 *R. raoultii* (MF166732, KX506737, JN398480), *Rickettsia* sp. (AH009131) | 3 | 100 | 3 *R. raoultii* (HQ232278, DQ365797, KU310593), *C.* Rickettsia rioja (GQ404431)* |

HD, Hohe Düne; TG, Torgelow; HN, Holländerei; UM, Ueckermünde; PZ, Putzar; n, number of isolates; *, isolates sequenced in both directions; n.a., not applicable.
